# Development of Anti-Virulence Approaches for Candidiasis via a Novel Series of Small-Molecule Inhibitors of *Candida albicans* Filamentation

**DOI:** 10.1128/mBio.01991-17

**Published:** 2017-12-05

**Authors:** Jesus A. Romo, Christopher G. Pierce, Ashok K. Chaturvedi, Anna L. Lazzell, Stanton F. McHardy, Stephen P. Saville, Jose L. Lopez-Ribot

**Affiliations:** a Department of Biology and South Texas Center for Emerging Infectious Diseases, The University of Texas at San Antonio, San Antonio, Texas, USA; b Department of Biology, University of the Incarnate Word, San Antonio, Texas, USA; c Department of Chemistry and Center for Innovation in Drug Discovery, The University of Texas at San Antonio, San Antonio, Texas, USA; Duke University

**Keywords:** *Candida albicans*, anti-virulence factor, antifungal drugs, filamentation, large-scale phenotypic screening

## Abstract

*Candida albicans* remains the main etiologic agent of candidiasis, the most common fungal infection and now the third most frequent infection in U.S. hospitals. The scarcity of antifungal agents and their limited efficacy contribute to the unacceptably high morbidity and mortality rates associated with these infections. The yeast-to-hypha transition represents the main virulence factor associated with the pathogenesis of *C. albicans* infections. In addition, filamentation is pivotal for robust biofilm development, which represents another major virulence factor for candidiasis and further complicates treatment. Targeting pathogenic mechanisms rather than growth represents an attractive yet clinically unexploited approach in the development of novel antifungal agents. Here, we performed large-scale phenotypic screening assays with 30,000 drug-like small-molecule compounds within ChemBridge’s DIVERSet chemical library in order to identify small-molecule inhibitors of *C. albicans* filamentation, and our efforts led to the identification of a novel series of bioactive compounds with a common biaryl amide core structure. The leading compound of this series, *N*-[3-(allyloxy)-phenyl]-4-methoxybenzamide, was able to prevent filamentation under all liquid and solid medium conditions tested, suggesting that it impacts a common core component of the cellular machinery that mediates hypha formation under different environmental conditions. In addition to filamentation, this compound also inhibited *C. albicans* biofilm formation. This leading compound also demonstrated *in vivo* activity in clinically relevant murine models of invasive and oral candidiasis. Overall, our results indicate that compounds within this series represent promising candidates for the development of novel anti-virulence approaches to combat *C. albicans* infections.

## IMPORTANCE

Since fungi are eukaryotes, there is a limited number of fungus-specific targets and, as a result, the antifungal arsenal is exceedingly small. Furthermore, the efficacy of antifungal treatment is compromised by toxicity and development of resistance. As a consequence, fungal infections carry high morbidity and mortality rates, and there is an urgent but unmet need for novel antifungal agents. One appealing strategy for antifungal drug development is to target pathogenetic mechanisms associated with infection. In *Candida albicans*, one of the most common pathogenic fungi, morphogenetic transitions between yeast cells and filamentous hyphae represent a key virulence factor associated with the ability of fungal cells to invade tissues, cause damage, and form biofilms. Here, we describe and characterize a novel small-molecule compound capable of inhibiting *C. albicans* filamentation both *in vitro* and *in vivo*; as such, this compound represents a leading candidate for the development of anti-virulence therapies against candidiasis.

## INTRODUCTION

*Candida albicans* is the main cause of opportunistic fungal infections in the expanding population of medically and immune-compromised patients (1, 2). Therapeutic options for the treatment of candidiasis are mostly restricted to azoles, polyenes, and echinocandins (3). Unfortunately, clinical use of these agents is severely limited due to their toxicity (mostly in the case of polyenes, such as amphotericin B) and the emergence of resistance (to azoles, but most recently also observed for echinocandins) (3–5), which contribute to high morbidity and mortality rates. In addition, candidiasis adds significant costs to our health care system. These limitations and poor outcomes highlight the urgent need for the development of novel antifungals, particularly those with new mechanisms of action (6, 7).

Fungi are eukaryotic, and as such have a reduced number of pathogen-specific targets that can be exploited for antifungal drug discovery. This is the main reason for the scarcity of antifungal drugs and also represents the main barrier to conventional antifungal drug development. An alternative strategy that has recently gained traction for antibiotic development is to target functions important for the pathogen’s virulence (6, 8, 9). This approach can be particularly attractive for fungal infections, because it immediately expands the number of potential targets (8). In the case of *C. albicans*, the implementation of such an anti-virulence approach could certainly benefit from the large body of research conducted during the last couple of decades that has provided important insights into the pathogenesis associated with these infections and has led to the identification of a number of virulence factors (10). Among *C. albicans* virulence factors, filamentation is perhaps the most important and, without any doubt, the one that has received the most attention to date. *C. albicans* can undergo morphogenetic transitions between yeast and filamentous morphologies, including hyphae and pseudohyphae, in response to different environmental stimuli (11, 12), likely reflecting the variety of conditions to which *C. albicans* needs to adapt during colonization and infection (13). At the molecular level, filamentation is tightly controlled through the activity of multiple complex signaling pathways, and different positive as well as negative regulators of filamentation have been identified (11, 12). After much speculation, the link between *C. albicans* filamentation and virulence is now firmly established. Many genetically defined mutant strains locked in terms of yeast morphology are nonpathogenic (10). Furthermore, experiments using the *C. albicans tet-NRG1* regulatable strain, in which morphogenetic conditions can be controlled both *in vitro* and *in vivo*, have provided compelling evidence for the requirement for filamentation in progression to the disease state and for the lethality associated with invasive candidiasis (14, 15). In addition, *C. albicans* is capable of forming biofilms, which enable the fungus in resisting antifungal treatment as well as evading attack from the host immune system (16). As such, biofilm formation greatly contributes to the pathogenicity of candidiasis (17). Importantly, biofilm formation and filamentation are intimately linked, as hyphae represent the main structural elements of mature biofilms and many key regulators of the *C. albicans* morphogenetic conversion also play a predominant role during the transition to the biofilm mode of growth (18–21).

We posited that filamentation represents a promising target for the development of a novel anti-virulence factor approach to combat *C. albicans* infections. We report here on a large-scale phenotypic screen that led to the identification of a novel series of biaryl amide small molecules, with the leading compound inhibiting filamentation under all growth conditions tested and displaying potent antibiofilm activity. Most significantly, we demonstrate this compound’s *in vivo* activity in clinically relevant murine models of invasive and oral candidiasis.

## RESULTS

### A large-scale phenotypic screening identified small-molecule inhibitors of *C. albicans* filamentation.

**FIG 1:**
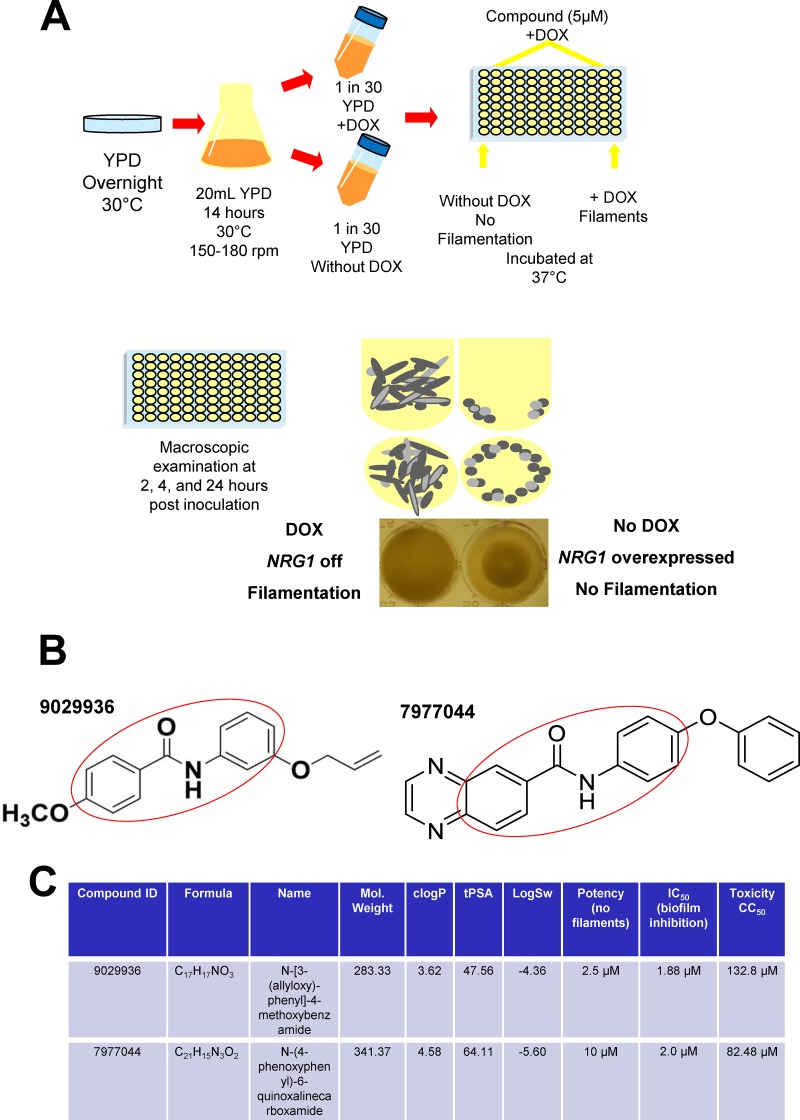
Primary screen to identify inhibitors of *C. albicans* filamentation. (A) Schematic diagram of the phenotypic assay used for large-scale phenotypic screening of 30,000 small-molecule compounds in the DIVERSet chemical library, which we used in our search for inhibitors of *C. albicans* filamentation. The screening uses 96-well round-bottom microtiter plates and takes advantage of tight control via doxycycline of morphogenetic conversions in the *C. albicans tet-NRG1* strain. Individual wells of the microtiter plates are seeded with fungal cells in the presence of 5 µM each individual compound, with appropriate positive and negative controls. The plates are incubated at 37°C and visually inspected at 2 h, 4 h, and 24 h. Under the conditions used, wells containing cells that grow the filamentous form (uninhibited by the presence of the compound) appear cloudy, whereas cells that grow in the yeast form (due to inhibition of filamentation in the presence of a hit compound) fall to the bottom of the wells and form rings, which are easily discernible macroscopically. Microscopy is then used for confirmation of the inhibitory effect on filamentation. (B) Chemical structures of compounds 9029936 and 7977044, two of the major initial hits identified in the primary screen. The two compounds share a common biaryl amide motif (highlighted in red). (C) Identity, physicochemical properties, including the clogP (partition coefficient and a measure of lipophilicity), tPSA (molecular polar surface area), and logSw (solubility of the drug in water), as well as IC_50_ (potency) and CC_50_ (toxicity) values, for these two small-molecule compounds.

As a first step in the development of anti-virulence approaches against candidiasis, we sought to identify compounds with inhibitory properties against *C. albicans* filamentation. To this end, we used a large-scale phenotypic assay to screen a set of 30,000 compounds from the DIVERSet chemical library (ChemBridge Corporation) for their ability to inhibit hypha formation. We developed a screening method for the inhibition of filamentation (Fig. 1A) that takes advantage of the tight control of morphogenesis in the genetically engineered *C. albicans tet-NRG1* strain previously developed in our laboratory (15). When this strain is grown in the absence of doxycycline, high *NRG1* expression levels block the yeast-to-hypha transition, whereas the presence of doxycycline inhibits the expression of the *tet*-*NRG1* allele, and filamentation is enabled. The screen was performed in 96-well round-bottom plates with yeast extract-peptose-dextrose (YPD) liquid medium at 37°C without serum and in the presence of doxycycline. In the case of the *tet-NRG1* strain, these growing conditions resulted in almost 100% filamentation. Individual compounds were tested at a final arbitrary concentration of 5 μM, a relatively low concentration, in order to avoid the high levels of background noise typically associated with screenings performed at higher concentrations, and the screening assays were performed in duplicate. Initial hits were easily identifiable visually: wells in which the compound inhibited filamentation contained a ring of settled cells (similar to the yeast control grown in the absence of doxycycline), as opposed to the cloudy appearance of wells in which filamentation was unimpeded by the presence of the test compound (Fig. 1A). Further confirmation of the inhibitory activity on filamentation of the initial hits was provided by microscopic observation of cells in the corresponding wells. Results from this screening identified a family of compounds with a common biaryl amide core structure, with compounds 9029936 and 7977044 displaying the highest levels of filamentation-inhibiting activity (Fig. 1B), and these two compounds were also predicted to display favorable “drug-like” characteristics according to their physico-chemical properties (Fig. 1C) (22). In addition, we determined the cytotoxicity levels of these compounds for a human hepatocyte cell line. As shown in Fig. 1C, the CC_50_ toxicity values (the concentration at which cellular viability was reduced by 50%) were high, in the range of 100 μM, likely indicating a good safety profile. Thus, 9029936 and 7977044 were designated our leading compounds for further characterization.

### Confirmation and further characterization of the inhibitory effects of the leading compounds on *C. albicans* filamentation.

**FIG 2:**
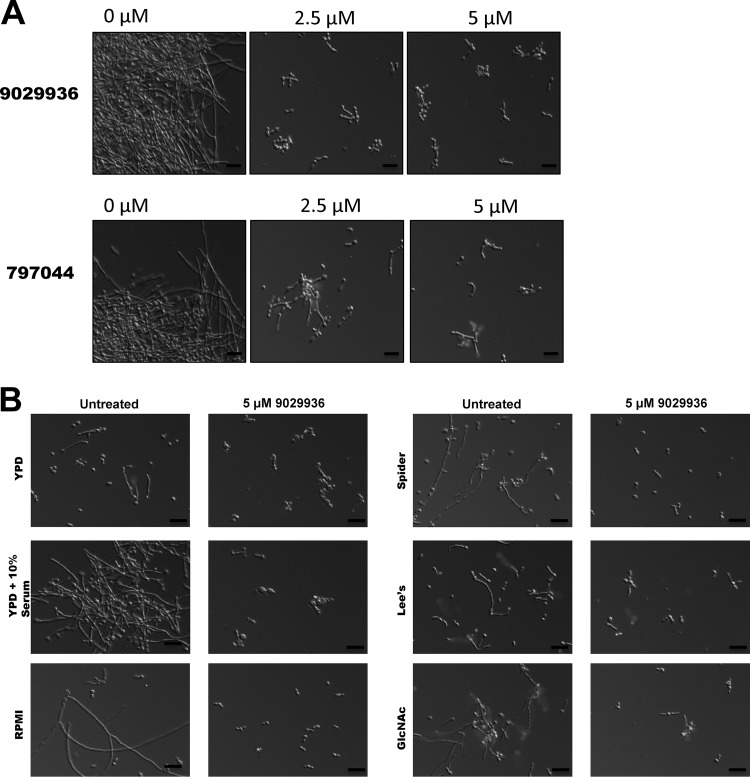
Inhibitory effects of the leading compounds on *C. albicans* filamentation. (A) Photomicrographs showing morphology of *C. albicans* strain SC5314 grown for 6 h under strong filament-inducing conditions (YPD plus serum, 37°C) in the presence of compounds 9029936 and 7977044. (B) Inhibition of filamentation in different liquid media. Liquid cultures of *C. albicans* strain SC5314 were grown in a variety of hypha-inducing media in the absence or presence of compound 9029936 at a concentration of 5 μM. Aliquots were taken from the different cultures at 6 h postinduction, visualized by DIC microscopy, and photographed. Bars, 20 μm.

In order to further confirm the antifilamentation properties of our leading compounds, we performed a series of dose-response assays using the same 96-well microtiter plate model, but this time we used the *C. albicans* wild-type strain SC5314 under strong filamentation-inducing conditions (YPD plus 10% fetal bovine serum [FBS] at 37°C). This is also important since it reaffirmed the efficacy of the leading compounds in the presence of serum, a critical requirement for activity *in vivo*. As seen in Fig. 2 (and also Fig. S1 in the supplemental material) and confirming results from our initial screen, almost complete inhibition of the yeast-to-hypha transition was observed at concentrations as low as 2.5 µM for compound 9029936 and 5 µM in the case of compound 7977044. Under non-filament-inducing conditions, treatment at these concentrations did not affect overall growth (Fig. S2), confirming that these small molecules behave as true anti-virulence factor compounds (although it should be noted that we did observe a modest effect on growth at higher concentrations [data not shown]).

10.1128/mBio.01991-17.1 FIG S1  Dose-dependent inhibitory effects of compound 9029936 (a) or 7977044 (b) on *C. albicans* filamentation. Photomicrographs show the morphologies of *C. albicans* strain SC5314 grown for 6 h in YPD plus serum at 37°C in the absence or presence of serial 2-fold dilutions of compound 9029936 (40 to 0.078 µM). Download FIG S1, PDF file, 0.1 MB. Copyright © 2017 Romo et al. 2017 Romo et al. This content is distributed under the terms of the Creative Commons Attribution 4.0 International license.

10.1128/mBio.01991-17.2 FIG S2  Compounds 9029936 and 7977044 do not affect overall growth of *C. albicans* yeast cells, confirming their anti-virulence mode of action. *C. albicans* SC5314 cells were seeded at an initial concentration of 0.5 × 10^3^ cells/ml into each well of round-bottom 96-well microtiter plates containing YPD, in the absence or presence of different concentrations (2.5 or 5 µM) of compounds 9029936 or 7977044. Plates were incubated at 30°C (non-filament-inducing conditions). Cell concentrations were assessed at 2, 4, 6, 8, 12, 18, 24, and 48 h by using a plate reader to determine the OD_600_, and the readings were used to generate the corresponding growth curves. Bars show standard deviations. Download FIG S2, PDF file, 0.2 MB. Copyright © 2017 Romo et al. 2017 Romo et al. This content is distributed under the terms of the Creative Commons Attribution 4.0 International license.

As mentioned previously, numerous environmental factors can induce yeast cells to filament through the action of several different and complex signaling pathways; this likely reflects the variety of microenvironments encountered by *C. albicans in vivo* (11–13). Therefore, we further examined the ability of compound 9029936 to inhibit filamentation in response to a variety of environmental cues stimulated under different growth conditions. Besides the response in the presence of serum, this leading compound blocked filamentation of *C. albicans* strain SC5314 during growth in *N*-acetyl glucosamine (GlcNAc; acting through Efg1) (12, 23, 24), Spider medium (acting through the cAMP pathway) (12, 25), Lee’s medium (acting through the Cph2 and Tec1 pathways) (12, 26), and RPMI 1640 medium (pH 7) (Fig. 2B); these represent the most common conditions used by a number of investigators in the field when inducing filamentation in liquid cultures. More recently, zinc chelation using diethylenetriamine pentaacetic acid (DTPA) has been shown to induce *C. albicans* filamentation under yeast growth conditions (i.e., YPD and 30°C) via the Hog1 pathway (27); compound 9029936 was also able to inhibit DTPA-induced filamentation at concentrations as low as 5 µM (Fig. S3), corroborating its potent inhibitory activity against multiple filamentation-inducing pathways.

10.1128/mBio.01991-17.3 FIG S3  Photomicrographs showing morphology of *C. albicans* strain SC5314 cells grown for 6 h in YPD in the presence of different concentrations of diethylenetriamine pentaacetic acid (DTPA) at 30°C (non-filament-inducing conditions) in the absence or presence of serial 2-fold dilutions of compound 9029936 (40 to 5 µM). Download FIG S3, PDF file, 0.2 MB. Copyright © 2017 Romo et al. 2017 Romo et al. This content is distributed under the terms of the Creative Commons Attribution 4.0 International license.

**FIG 3:**
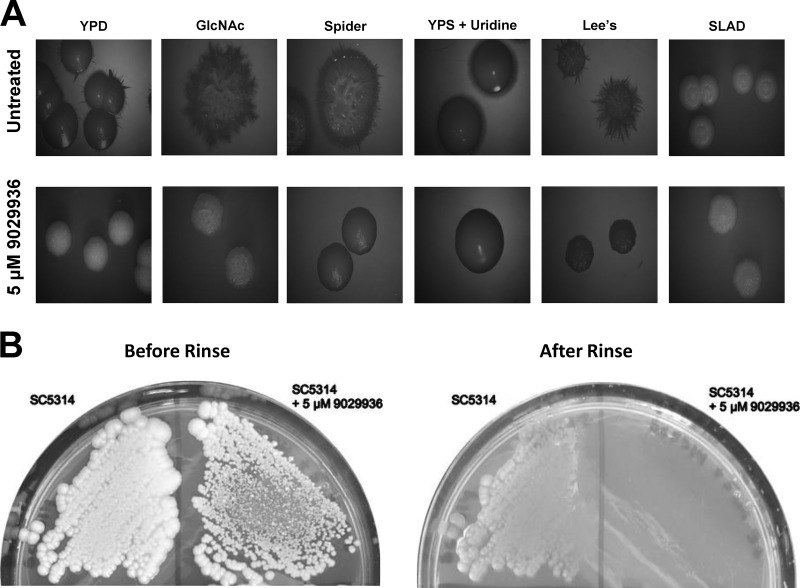
Inhibitory effects of leading compound 9029936 on *C. albicans* filamentation on solid media. (A) Colony morphologies of *C. albicans* strain SC5314 grown on various solid filament-inducing media in the absence or presence of compound 9029936 at 5 μM. Plates were incubated at 37°C for 5 days. A wrinkled colony morphology indicates the presence of filamentous cells. (B) Compound 9029936 inhibits *C. albicans* agar invasion. *C. albicans* SC5314 cells were streaked on YPD plates with or without compound 9029936 at 5 μM, and the plates were incubated at 37°C for 5 days. The plates were photographed prior to (left) and after (right) surface cells were removed by gentle washing under running water. In the presence of the compound, cells grew mostly on the surface of the medium and were washed away, whereas cells grown in the absence of the compound had invaded the agar and remained after washing.

Likewise, as shown in Fig. 3A, *C. albicans* SC5314 formed wrinkled colonies on a variety of solid media; however, when compound 9029936 at 5 µM was added to the plates, smooth colonies were observed on YPD, Lee’s pH 7 (26), Spider (25), and synthetic low-ammonium–dextrose (SLAD) media (28, 29), as well as on yeast extract-peptone-sucrose (YPS) medium plus uridine (mimicking embedded conditions) (30–32). Although wrinkled colonies were still observed on GlcNAc plates, we noted a lack of invasive filamentous growth into the agar when SC5314 was grown in the presence of this compound. Furthermore, in a “typical” agar invasion assay, streaked cells grown in the presence of compound 9029936 washed away easily, compared to uninhibited cells that invaded the agar (Fig. 3B). Similar effects were observed in the case of compound 7977044 under both liquid and solid conditions (Fig. S4).

10.1128/mBio.01991-17.4 FIG S4  (A) Inhibition of filamentation by compound 7977044 in different liquid media. Liquid cultures of *C. albicans* strain SC5314 were grown in a variety of hypha-inducing media in the absence or presence of compound 7977044 at a concentration of 5 µM. Samples were retrieved from different cultures 6 h postinduction, visualized by DIC microscopy, and photographed. Bars, 20 μm. (B) Colony morphologies of *C. albicans* strain SC5314 grown on various solid filament-inducing media in the absence or presence of compound 7977044 at 5 µM. Plates were grown at 37°C for 5 days. A wrinkled colony morphology indicates the presence of filamentous cells. Download FIG S4, PDF file, 0.2 MB. Copyright © 2017 Romo et al. 2017 Romo et al. This content is distributed under the terms of the Creative Commons Attribution 4.0 International license.

**FIG 4:**
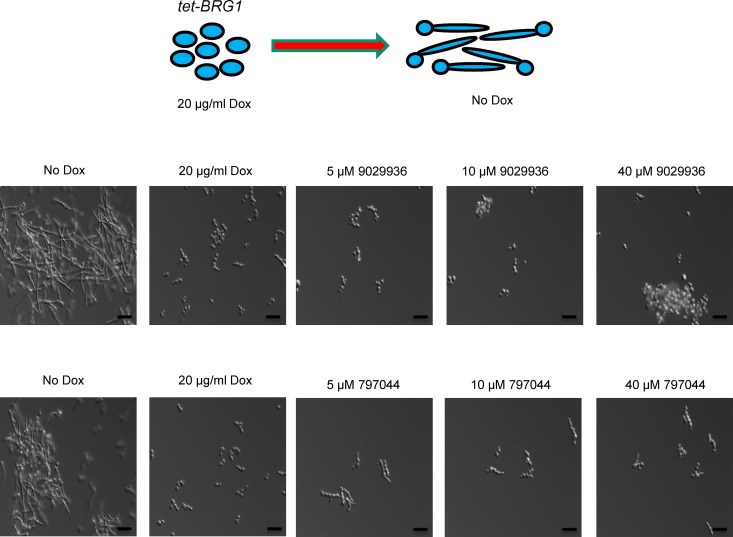
Compounds 9029936 and 7977044 inhibit *C. albicans* filamentation that is driven by overexpression of *BRG1*. In the absence of doxycycline, overexpression of *BRG1* in the *C. albicans tet-BRG1* strain led to filamentation under noninducing conditions (YPD medium at 30°C, no serum). Addition of compound 9029936 or 7977044 led to inhibition of filamentation of this strain under these conditions.

Overall, these results indicated that compound 9029936 inhibits filamentation under numerous inducing conditions, strongly suggesting that it impacts a common, core component of the cellular machinery that mediates hypha formation, most likely a component downstream of multiple different signaling pathways. This is very reminiscent of the phenotype described previously for the *C. albicans Δbrg1* mutant strain (33, 34). Our group recently demonstrated that in a tetracycline-regulatable *C. albicans tet-BRG1* strain, overexpression of *BRG1* (when the strain is grown in the absence of doxycycline) drives filamentation even under noninducing conditions (i.e., YPD at 30°C) (35). Thus, we tested the abilities of compounds 9029936 and 7977044 to inhibit filamentation of this strain (Fig. 4). As expected, in the absence of these compounds, the *C. albicans tet-BRG1* strain was able to filament when doxycycline was not present and remained in the yeast form in the samples containing the tetracycline derivative. However, the presence of compound 9029936 or 7977044 at concentrations as low as 2.5 µM inhibited filamentation of the *C. albicans tet-BRG1* strain when it was grown without doxycycline. Overall, these results point to a potential mechanism of action downstream of multiple signal transduction pathways and most likely impacting Brg1 regulation of filamentation.

### The leading compounds inhibited *C. albicans* biofilm formation.

**FIG 5:**
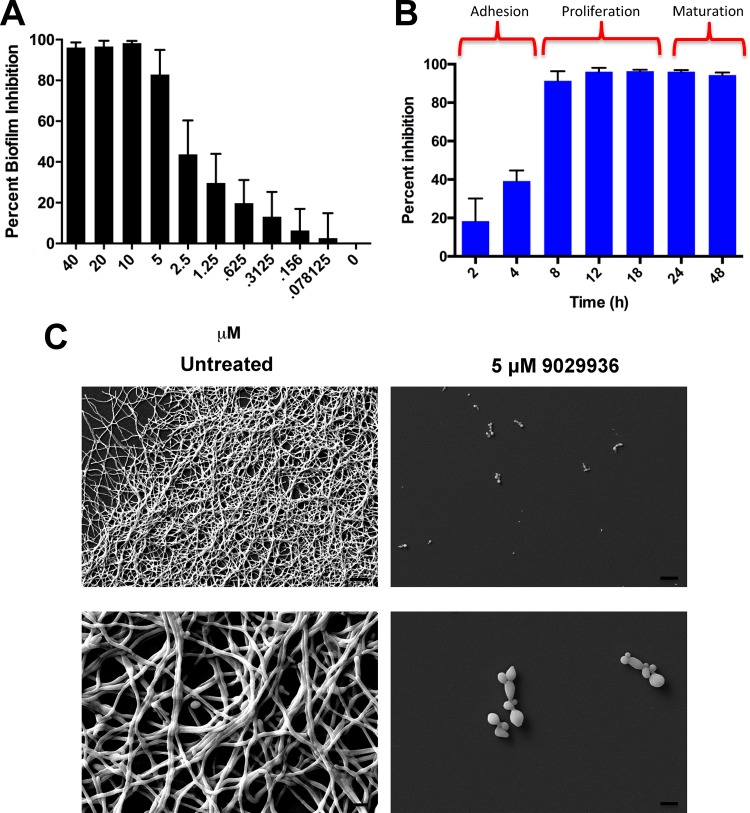
*In vitro* characterization of the inhibitory activity of the leading compound 9029936 on *C. albicans* biofilm formation. (A) Dose-dependent inhibitory effects of compound 9029936 on *C. albicans* biofilm formation. The compound was tested in serial 2-fold dilutions (concentrations ranging from 40 to 0.078 µM), with appropriate positive and negative controls. Results shown are the mean percent biofilm inhibition relative to control biofilms (grown in the absence of compound 9029936), determined in XTT colorimetric assays for multiple technical replicates from several independent experiments. Error bars indicate standard deviations. (B) The leading compound inhibited the proliferation and maturation phases of *C. albicans* biofilm development. A biofilm kinetic assay was performed in order to examine the inhibitory effects of compound 9029936 at different stages of biofilm development, using the same 96-well microtiter plate model of *C. albicans* biofilm formation. The extent of inhibition was determined at multiple times (2, 6, 8, 12, 24, and 48 h) after seeding the wells with *C. albicans* cells in the presence or absence of compound 9029936 (5 μM concentration). Results are expressed as means of multiple technical replicates from a single experiment, with error bars representing standard deviations. (C) SEM images of biofilms of *C. albicans* strain SC5314 formed in the absence or presence of compound 9029936 at a concentration of 5 μM. Bars, 20 μm.

The ability to form biofilms represents another major virulence factor associated with *C. albicans* infections, and this process is intimately linked with filamentation (8, 16). Compounds 9029936 and 7977044 were shown to be potent inhibitors of biofilm formation by *C. albicans* strain SC5314 in the standard 96-well microtiter plate model previously developed by our laboratory (36, 37) (Fig. 5A; Fig. S5 and S6), with calculated IC_50_s (the concentration of each compound resulting in 50% inhibition of biofilm formation) of 1.875 μM and 2.006 μM, respectively. Interestingly, both compounds retained some level of activity against preformed biofilms, although we noted that this effect was observed at higher concentrations, at which these compounds exerted a more general antifungal effect (Fig. S7). Figure S8 also shows how the leading compound 9029936 displayed potent inhibitory activity against *C. albicans* biofilm formation on silicone, a more clinically relevant model for catheter-related biofilm formation. A biofilm kinetic assay indicated that compound 9029936 exerted its inhibitory activity mostly during the proliferation and maturation phases of biofilm formation and not during the early adhesion phase (Fig. 5B); similar observations were made with compound 7977044 (Fig. S5B). These observations are consistent with the inhibitory activities of the compounds against filamentation, since early adhesion is mediated by yeast cells but filamentation represents a critical step, particularly during the intermediate proliferation phase of *C. albicans* biofilm development. Scanning electron microscopy (SEM) demonstrated that incubation in the presence of compound 9029936 led to a rather poor biofilm that consisted mostly of sparsely attached yeast cells, compared to the typically robust and highly filamentous control biofilms that formed in the absence of compound (Fig. 5C).

10.1128/mBio.01991-17.5 FIG S5  (A) Dose-dependent inhibitory effects of compound 7977044 on *C. albicans* biofilm formation, as revealed in an XTT colorimetric assay. The compound was tested in serial 2-fold dilutions at concentrations ranging from 40 to 0.078 µM, with appropriate positive and negative controls. Results shown are the percent biofilm inhibition relative to control biofilms (in the absence of compound). (B) Compound 7977044 inhibits the proliferation and maturation phases of *C. albicans* biofilm development. A biofilm kinetic assay was performed in order to examine the inhibitory effects of compound 7977044 at different stages of biofilm development, using the same 96-well microtiter plate model of *C. albicans* biofilm formation. The extent of inhibition was determined at multiple times (2, 6, 8, 12, 24, and 48 h) after seeding the wells with *C. albicans* cells in the presence or absence of compound 7977044 (5 µM concentration). Experiments were performed in duplicate with multiple replicates in each test, and bars show standard deviations. Download FIG S5, PDF file, 0.2 MB. Copyright © 2017 Romo et al. 2017 Romo et al. This content is distributed under the terms of the Creative Commons Attribution 4.0 International license.

10.1128/mBio.01991-17.6 FIG S6  Dose-dependent inhibitory effects of compounds 9029936 and 7977044 on *C. albicans* biofilm formation, as revealed by crystal violet (CV) staining. Each compound was tested in serial 2-fold dilutions at concentrations ranging from 40 to 0.078 µM, with appropriate positive and negative controls. Results shown are the percent biofilm inhibition relative to control biofilms (in the absence of compound); the CV assay allowed estimation of total biofilm biomass (as opposed to an XTT assay, which measures metabolic activity of cells within a biofilm). Experiments were performed in duplicate with multiple replicates in each test; bars show standard deviations. Download FIG S6, PDF file, 0.3 MB. Copyright © 2017 Romo et al. 2017 Romo et al. This content is distributed under the terms of the Creative Commons Attribution 4.0 International license.

10.1128/mBio.01991-17.7 FIG S7  Dose-dependent inhibitory activities of compounds 9029936 and 7977044 against preformed biofilms. *C. albicans* SC5314 biofilms were formed in microtiter plates for 24 hours as described elsewhere (in the absence of compound). The plates were then washed, and the individual compounds were added to each test well in serial 2-fold dilutions ranging from 40 to 0.078 µM. The microtiter plates were then incubated at 37°C for an additional 24 h and processed using the XTT colorimetric assay. Experiments were performed in duplicate with multiple replicates in each test, and bars show standard deviations. Download FIG S7, PDF file, 0.1 MB. Copyright © 2017 Romo et al. 2017 Romo et al. This content is distributed under the terms of the Creative Commons Attribution 4.0 International license.

10.1128/mBio.01991-17.8 FIG S8  Inhibition of biofilm formation on silicone. Biofilms were formed on silicone in the presence or absence of different concentrations of compound 9029936. The percent inhibition was determined in an XTT reduction assay. Experiments were done in duplicate with multiple replicates in each test, and results are expressed as means and standard deviations. Download FIG S8, PDF file, 0.03 MB. Copyright © 2017 Romo et al. 2017 Romo et al. This content is distributed under the terms of the Creative Commons Attribution 4.0 International license.

### *In vivo* efficacies of the leading compounds in clinically relevant murine models of invasive and oral candidiasis.

**FIG 6:**
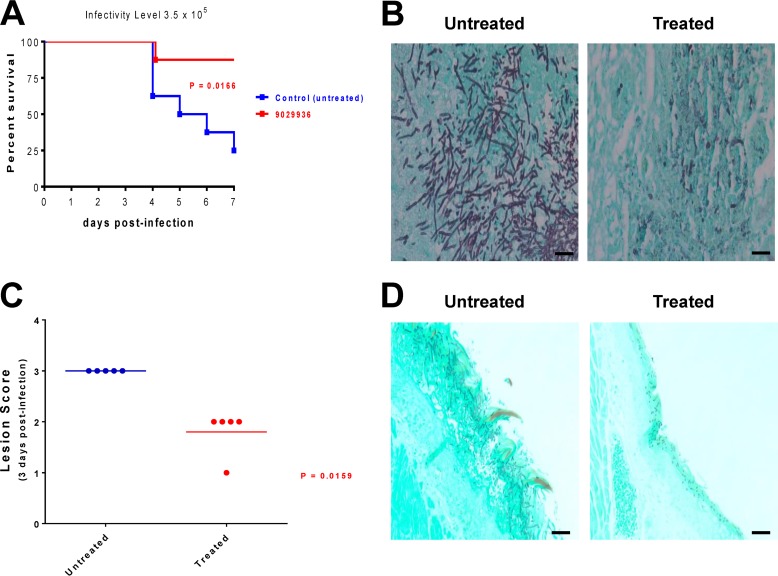
*In vivo* activity of the leading compound 9029936 in the clinically relevant murine models of hematogenously disseminated invasive candidiasis and oropharyngeal candidiasis. (A) Protection in the murine model of hematogenously disseminated invasive candidiasis. Survival curves show results for a group of mice treated with compound 9029936 compared to an untreated control group. By the end of the experiment, 87.5% of animals that received treatment with compound 9029936 survived the infection, resulting in statistically significant differences versus the control group. (B) Histological analyses of kidney sections retrieved from untreated mice 3 days after intravenous infection with *C. albicans* strain SC5314 showed typical kidney lesions of a principally filamentous nature, whereas isolated cells or groups of cells with a mostly yeast morphology were predominant in kidneys recovered from mice treated with compound 9029936 at the same time point. Bars, 20 μm. (C) Efficacy of compound 9029936 against oral candidiasis in mice, as assessed by clinical score. Mice were immunosuppressed and infected orally with *C. albicans* strain SC5314. One group of mice received vehicle only and served as untreated controls, while another group of mice received treatment with compound 9029936. Mice were monitored for signs of oral candidiasis and assigned a clinical score on the basis of the extent and severity of tongue lesions. The graph depicts clinical scores for mice sacrificed 3 days after infection, for which there were statistically significant differences between treated and control mice. (D) Histological analyses revealed an abundant biofilm composed mostly of filamentous cells covering the tongue surface and penetrating deep into the tissues of untreated animals, whereas fewer cells, displaying mostly an elongated yeast morphology and superficially located, were detected in tongues from mice treated with the leading compound 9029936. Bars, 20 μm.

After confirming the ability of our leading compounds to inhibit filamentation and biofilm formation *in vitro*, as well as revealing a relatively safe profile through toxicity studies, we proceeded to examine their efficacy in the clinically relevant murine models of hematogenously disseminated candidiasis (15, 38, 39) and oropharyngeal candidiasis (40, 41). In the hematogenously disseminated model, seven of eight animals that received treatment with compound 9029936 survived the infection, compared to results in the untreated group, in which six of eight animals succumbed to the infection by day 7 (Fig. 6A); these differences were statistically significant (*P* = 0.0166). Consistent with an inhibition of the filamentation mechanism, histological examination of kidneys from untreated animals indicated that the fungal cells infecting the tissues displayed a highly filamentous morphology, whereas the kidneys from treated animals contained mostly scattered yeast cells (Fig. 6B). Similar results were obtained in the case of compound 7977044 (Fig. S9). Also reinforcing its anti-virulence mode of action, no major differences in fungal burdens were observed between animals treated with compound 9029936 and untreated mice when animals were sacrificed at different times postinfection (Fig. S10); however, we acknowledge that filaments may have a lower plating efficiency than yeast cells, and therefore fungal burdens in organs from untreated mice may be underestimated.

10.1128/mBio.01991-17.9 FIG S9  *In vivo* activity of compound 7977044 in a murine model of hematogenously disseminated candidiasis. (A) Protection in the murine model of hematogenously disseminated invasive candidiasis. Compound 7977044 was administered to a group of mice once daily by intraperitoneal injection (20 mg/kg) starting 2 days prior to intravenous infection with *C. albicans* strain SC5314. A control (untreated) group of mice received vehicle-only injections. Treatment continued for 7 days postinfection, and survival curves were determined and analyzed using the Kaplan-Meier and log rank tests. By the end of the experiment, 75% of animals that received treatment with compound 9029936 survived the infection, resulting in statistically significant differences versus the control group (*P* = 0.0272). (B) Histological analyses (GMS staining) of kidney sections retrieved from untreated mice 3 days after i.v. infection with *C. albicans* strain SC5314 showed typical kidney lesions of a principally filamentous nature, whereas isolated cells or groups of cells with a mostly yeast morphology were predominant in kidneys recovered from mice treated with compound 7977044 at the same time point. Bar, 40 μm (10× panel) or 20 μm (40× panel). Download FIG S9, PDF file, 0.2 MB. Copyright © 2017 Romo et al. 2017 Romo et al. This content is distributed under the terms of the Creative Commons Attribution 4.0 International license.

10.1128/mBio.01991-17.10 FIG S10  In a model of invasive candidiasis, kidney fungal burdens were similar in control (untreated) animals and in animals treated with compound 9029936. One group of mice was treated with compound 9029936 (20 mg/kg, injected intraperitoneally once daily starting 2 days prior to infection), whereas another group (untreated control) was injected with vehicle only. All animals were infected with 2.5 × 10^5^ *C. albicans* SC5314 cells, and four animals from each group were then sacrificed at 1, 2, 3, and 6 days postinfection, at which time the fungal loads in kidneys were determined. Consistent with an anti-virulence compound, there were no statistically significant differences in fungal organ loads in treated versus untreated animals (analyzed using the Mann-Whitney test). Similar results were found for brain and spleen fungal burdens (data not shown). Download FIG S10, PDF file, 0.01 MB. Copyright © 2017 Romo et al. 2017 Romo et al. This content is distributed under the terms of the Creative Commons Attribution 4.0 International license.

In the oropharyngeal model of candidiasis, the severity of lesions and associated clinical scores were decreased in treated animals compared to control (untreated) animals (Fig. 6C). Validating these results, histopathological examination of the tongues of untreated mice demonstrated a widespread hyphal biofilm on the surface and penetrating the epithelium, whereas only superficially located yeast cells were present on tongues from mice treated with compound 9029936 (Fig. 6D). Similar results were obtained in the case of compound 7977044 (results not shown). Importantly, these results point to the efficacy of an anti-virulence approach even under immune-suppressive conditions.

## DISCUSSION

The unacceptably high morbidity and mortality rates associated with *C. albicans* infections highlight the urgent need for the development of novel therapeutic approaches to complement the exceedingly small arsenal of antifungal agents currently available for the treatment of candidiasis (6, 7). A plethora of studies by multiple groups of investigators has provided important insights into the pathogenesis of candidiasis (10), and this information should represent an optimal starting point for the translation of these findings into strategies that can directly benefit patients suffering from these devastating infections. Chief among these should be the development of novel anti-virulence approaches for the treatment of candidiasis (8). Because filamentation constitutes the major virulence factor during *C. albicans* infections, it represents an attractive yet clinically unexploited target for the development of such alternative anti-virulence strategies (42). Our increased knowledge of *C. albicans* filamentation at the molecular level and the identification of several molecules that inhibit the yeast-to-hypha transition (at least *in vitro*), however, have not yet crystallized into the development of any new antifungal agents for the treatment of these infections (8, 42–44). Here, using phenotypic screening techniques, we identified a novel family of small-molecule compounds with a common biaryl amide core structure that represent potent inhibitors of *C. albicans* filamentation. The leading compound of this series prevented filamentation under all conditions tested, suggesting that it impacts an important node that lies downstream from several, if not all, of the major different signaling pathways controlling the yeast-to-hypha transition; this is likely to be important given the complexity and redundancy of the circuitry controlling filamentation. Perhaps not surprisingly, given the intimate link between filamentation and biofilm formation, subsequent experiments also confirmed the antibiofilm activity of compounds within this series. Importantly, the *in vivo* activity of the leading compounds in clinically relevant models of invasive and oral candidiasis, together with reasonable safety profiles, further reinforce their potentials as promising candidates in the development of a novel anti-virulence approach. The implementation of such a strategy is particularly appealing due to the fact that it immediately and significantly expands the repertoire of potential targets for antifungal drug discovery and development. Indeed, the scarcity of conventional targets is the main reason behind both the limited existing armamentarium of antifungal agents and the toxicity displayed by some of these medications (6). One additional significant advantage of anti-virulence approaches is that they do not kill or arrest growth of the pathogen. Rather, they disarm the microorganism from its pathogenic potential, and therefore they impose a much weaker selective pressure for the development of resistance (45), which also constitutes a major problem, particularly with regard to the azoles and echinocandins (46). Moreover, as a normal commensal of humans, *C. albicans* is ideally suited to such a nonlethal treatment option. One caveat is that, by definition, these anti-virulence approaches exhibit a narrower window of activity and spectrum of action and are only effective against the species displaying the specific virulence trait (45), in this case, only *C. albicans* and not other *Candida* species (perhaps with the exception of the closely related *C. dubliniensis*) (47). As such, their future usage will necessarily rely on our ability to accurately diagnose the infection.

In conclusion, as the emergence of resistance poses increasing threats to our ability to successfully treat these infections and the need for new antifungal drugs continues to grow, the implementation of anti-virulence approaches represents a compelling strategy, and a new paradigm, for the discovery and development of new antifungal drugs with novel modes of action (8, 42). This study provides evidence for the efficacy of such anti-virulence approaches against *C. albicans* infections, and a similar strategy may be applicable in the future to other fungal pathogens, as well as to other disease-causing mechanisms besides filamentation and biofilm formation. We are currently embarked on a medicinal chemistry campaign to improve the pharmacological characteristics of the leading compounds and are also attempting to identify their specific molecular target, as prerequisites for their further preclinical and clinical development.

## MATERIALS AND METHODS

### Strains, media, and culture conditions.

The wild-type *C. albicans* strain SC5314 as well as the genetically engineered *C. albicans tet-NRG1* and *tet-BRG1* doxycycline regulatable mutant strains (15, 35) were utilized for these studies. Cell stocks were stored at −80°C and propagated by streaking onto YPD agar plates (1% [wt/vol] yeast extract, 2% [wt/vol] peptone, 2% [wt/vol] dextrose, and 1.5% agar) and incubated overnight at 30°C. From these, a loopful of cells was inoculated into flasks (150 ml) containing 25 ml of YPD liquid medium in an orbital shaker at 180 rpm and grown overnight for 14 to 16 h at 30°C. Under these conditions, *C. albicans* grows as a budding yeast. The *C. albicans tet-BRG1* strain was grown overnight in the presence of 20 µg/ml of doxycycline to prevent filamentation.

### Chemical library.

A total of 30,000 small molecules from the DIVERSet library from ChemBridge Corporation (San Diego, CA) were screened. Compounds in this library are novel, diverse, and easy to follow up on. Furthermore, these small molecules generally have favorable pharmacological properties and meet very stringent drug-like properties. Compounds in the library were provided as stock solutions at 10 mM in dimethyl sulfoxide (DMSO) in 96-well microtiter bar-coded plates. Before screening, the compounds were diluted to 0.1 mM by pipetting 2 μl of the concentrated solution into 198 μl of sterile phosphate-buffered saline (PBS; 10 mM phosphate buffer, 2.7 mM potassium chloride, 137 mM sodium chloride, pH 7.4 [Sigma, St. Louis, MO, USA]) using the wells of presterilized, polystyrene, round-bottom, 96-well microtiter plates (Corning Inc., Corning, NY) and stored as working stock solutions at −20°C. For follow-up experiments, milligram quantities were obtained from stock compounds available for hit resupply from ChemBridge Corporation.

### Screening for inhibitors of *C. albicans* filamentation.

The screening method in our search for inhibitors of filamentation took advantage of the tight control of morphogenesis in the genetically engineered C. albicans *tet-NRG1* strain previously developed in our laboratory (15). When this strain is grown in the absence of doxycycline, high *NRG1* expression levels block the yeast-to-hypha transition, whereas the presence of the antibiotic inhibits the expression of the *tet*-*NRG1* allele and thus enables filamentation. The screen is performed in 96-well round-bottom plates in YPD liquid medium at 37°C without serum and in the presence of doxycycline. In the case of the *tet-NRG1* strain, these growing conditions resulted in almost 100% filamentation. Individual wells of the microtiter plates were seeded with fungal cells (by preparing a 1 in 30 dilution from an overnight culture) in the presence of a 5 µM concentration of each individual compound (a total of 30,000 compounds were tested individually), with appropriate positive and negative controls. The plates were incubated at 37°C and visually inspected at 2 h, 4 h, and 24 h postinduction. The initial screen was performed in duplicate. Under the conditions used, wells containing cells that grew in the filamentous form, which was uninhibited by the presence of the compound, appeared cloudy, whereas cells that grew in the yeast form, due to inhibition of filamentation in the presence of a hit compound, fell to the bottom of the wells and formed rings, which were easily discernible macroscopically. Further confirmation of the inhibitory activity on filamentation of initial hits was provided by microscopic observation of cells in the corresponding wells.

### Dose-response assays for effects on *C. albicans* filamentation.

*C. albicans* strain SC5314 was grown overnight as described above, washed with PBS, and used to seed a round-bottom 96-well microtiter plate with YPD containing 10% FBS at a 1:30 dilution. Compounds 9029936 and 7977044 were each serially diluted, starting at 40 μM and ending at 0.078 μM. Plates were incubated for 6 h at 37°C and examined microscopically for morphological differences between treated cells and controls. Microscopy was performed using differential interference contrast (DIC) on an Axio observer D1 inverted microscope (Carl Zeiss, Inc., Thornwood, NY) equipped for photography.

### Inhibition of filamentation under different growth medium conditions.

In order to further elucidate the mechanism of action of compound 9029936, different growth conditions able to stimulate filamentation were utilized. Medium conditions selected included YPD, YPD plus 10% FBS, RPMI 1640, Lee’s (26), Spider (25), GlcNAc (23), SLAD (28, 29), and YPS plus uridine (30). Growth of *C. albicans* SC5314 was tested on both solid (YPD, Lee’s, Spider, GlcNAc, SLAD, and YPS plus uridine) and liquid (YPD, YPD plus 10% FBS, RPMI, Lee’s, Spider, and GlcNAc) media. For growth on solid media, cells from a 2 × 10^6^ cells/ml solution were serially diluted and plated in the absence and presence of compound 9029936 or 7977044 at a concentration of 5 µM. Plates were incubated at 37°C for 5 to 7 days. Individual colonies were then photographed using a stereomicroscope. For liquid conditions, cells from an overnight culture were added to medium at a 1:30 dilution, washed with PBS, and incubated at 37°C for 24 h. Microscopy was performed using DIC on an Axio Observer D1 inverted microscope (Carl Zeiss, Inc., Thornwood, NY) equipped for photography.

### Invasion assays.

For invasion assays, compound 9029936 was added to half of the plate at a concentration of 5 μM. Plates were then placed at 30°C to allow for compound diffusion. A 1:1,000 dilution of *C. albicans* SC5314 was made from a 2 × 10^6^ cells/ml solution and streaked onto a quadrant with compound 9029936 and onto a quadrant without compound. Plates were incubated at 37°C for 5 to 7 days. Images were taken before and after rinsing plates with water.

### DTPA assays to induce filamentation.

*C. albicans* SC5314 cells were used to seed a round-bottom microtiter plate containing a combination of compound 9029936 and DTPA (Sigma-Aldrich, St. Louis, MO) in YPD. In a checkerboard manner, serial 2-fold dilutions of compound 9029936 (ranging from 40 to 0.156 µM) and serial 2-fold dilutions of DTPA (ranging from 100 to 1.5 µM) were added to the wells of 96-well round-bottom microtiter plates in combination before seeding with aliquots of a *C. albicans* SC5314 culture in YPD (100 µl/well of a 1 × 10^6^ cells/ml solution). An appropriate positive control (absence of compound, to allow for uninterrupted filamentation) and negative control (no cells, to monitor contamination) were added. The plates were incubated at 30°C for 6 h to allow for filamentation. Microscopy was performed using DIC on an Axio Observer D1 inverted microscope (Carl Zeiss, Inc., Thornwood, NY) equipped for photography.

### Inhibitory effect on filamentation of the *C. albicans tet-BRG1* strain.

The *C. albicans tet-BRG1* strain (35) was grown overnight as described above, washed with PBS, and used to seed a round-bottom 96-well microtiter plate with YPD at a 1:30 concentration. Serial 2-fold dilutions of compound 9029936 or 7977044 starting at 40 μM and ending at 2.5 μM were prepared. Columns with doxycycline and no compound (inhibition of filamentation), as well as no doxycycline and no compound (extensive filamentation) were added as controls. Plates were incubated at 30°C for 6 h. Microscopy was performed using DIC on an Axio Observer D1 inverted microscope (Carl Zeiss, Inc., Thornwood, NY) equipped for photography.

### *C. albicans* growth curves under noninducing conditions.

Growth curves were generated using a 96-well microtiter plate with round-bottom wells. *C. albicans* SC5314 cells were seeded into each well containing YPD at a concentration of 0.5 × 10^3^ cells/ml in the absence or presence of 9029936 or 7977044 at concentrations of 2.5 and 5 µM. Plates were incubated at 30°C. Cell concentrations were assessed at 2, 4, 6, 8, 12, 18, 24, and 48 h by using a plate reader (which measured the optical density at 600 nm [OD_600_]).

### Effects on *C. albicans* biofilm formation and preformed biofilms.

The *C. albicans* biofilm formation assays used the 96-well flat-bottom plate model originally developed by our group (36, 37). For inhibition of biofilm formation, serial 2-fold dilutions (ranging from 40 to 0.078 µM) of compound 9029936 or 7977044 were added to the wells of 96-well flat-bottom microtiter plates before seeding with aliquots of a *C. albicans* SC5314 culture in RPMI 1640 medium supplemented with l-glutamine (Corning) and buffered with 165 mM morpholinepropanesulfonic acid (MOPS; Sigma; 100 µl/well of a 1 × 10^6^ cells/ml solution). Appropriate positive (absence of drug, to allow for uninterrupted biofilm formation) and negative (no cells, to monitor contamination and to be able to calculate the percent inhibition) controls were included. Each compound was assayed at least in duplicate plates (biological replicates), with 4 to 8 technical replicates per plate. The plates were incubated at 37°C for 24 h to allow for biofilm formation. After the incubation period, the plates were washed twice with 200 µl/well of PBS, and biofilm inhibition was measured in a 2,3-bis-(2-methoxy-4-nitro-5-sulfophenyl)-2H-tetrazolium-5-carboxanilide (XTT) colorimetric assay. The IC_50_, defined as the concentration of each compound leading to 50% inhibition of biofilm formation, was calculated from the results of these assays, via use of the Prism program (GraphPad Software, Inc., San Diego, CA). In addition, for the examination of activity against preformed biofilms, *C. albicans* SC5314 biofilms were formed in microtiter plates during 24 hours as described before (in the absence of compound); the plates were then washed twice with sterile PBS, and 100 µl of RPMI containing individual compounds in serial 2-fold dilutions was added to each test well. The microtiter plates were then incubated at 37°C for an additional 24 h, washed twice with sterile PBS, and processed for the XTT reduction assay.

A similar series of experiments was performed in which biofilms were stained with crystal violet to assess biofilm biomass, as previously described (48). Briefly, after the last incubation, plates were washed twice with PBS and each well was treated with 100 µl of methanol for 20 min for fixation. Methanol was removed and plates allowed to dry. Adherent biofilms were then stained for 10 min with 150 µl of 3% (wt/vol) crystal violet. After crystal violet was removed and plates were allowed to dry, they were washed thrice with 200 µl of distilled water. Microscopy was performed using a light microscope. For each well, 100 µl of 33% glacial acetic acid was used to dissolve the dye (after microscopy). Glacial acetic acid was left in the wells for 5 min while plates were shaken slowly. Solutions were then transferred to a new microtiter plate for OD_550_ measurements to calculate the extent of biofilm inhibition compared to untreated controls.

### Effects on biofilm formation on silicone elastomers.

Silicone elastomer sheets (Bentec Medical, Woodland, CA) were cut into 1-cm by 1-cm squares, cleaned by washing with hand soap and water, and autoclaved (49). Prior to biofilm formation, the silicone coupons were incubated in fetal bovine serum overnight at 37°C. The pieces were washed twice in sterile PBS and placed into the wells of 24-well flat-bottom presterilized microtiter plates (Corning). Then, 2 ml of a 5 × 10^6^ cels/ml suspension of *C. albicans* SC5314 in RPMI buffered with MOPS containing either the leading compound at final concentrations of 2.5, 5, 10, and 20 µM or no compound (control) was added to the wells containing the silicone pieces and incubated in an orbital shaker at 100 rpm for 2 h at 37°C. Following this adhesion step, nonadherent cells were removed by washing the silicone pieces twice in sterile PBS and transferring them to wells of a new 24-well plate. Fresh RPMI medium containing the compound at the same concentrations was added to the corresponding wells, and the plates were incubated for an additional 24 h in an orbital shaker (100 rpm) at 37°C. After this incubation, the silicone pieces were washed with PBS and processed for the XTT reduction assay to calculate the percent inhibition. For these experiments, each compound was assayed in duplicate wells of a microtiter plate.

### Kinetic assays to determine inhibitory effects at different stages of biofilm development.

For biofilm kinetic assays, serial 2-fold dilutions (ranging from 40 to 0.078 µM) of 9029936 or 7977044 were added to the wells of 96-well flat-bottom microtiter plates before seeding with aliquots of a *C. albicans* SC5314 culture in RPMI 1640 medium supplemented with l-glutamine (Corning) and buffered with 165 mM morpholinepropanesulfonic acid (100 µl/well of a 1 × 10^6^ cells/ml solution). Appropriate positive and negative controls were added. The plates were incubated at 37°C for 2, 4, 8, 12, 18, 24, or 48 h to allow for biofilm formation. After each incubation period, the plates were washed twice with 200 µl/well of PBS, and biofilm inhibition was measured in an XTT colorimetric assay. For these experiments, each compound was assayed in duplicate plates, with 4 to 8 technical replicates per plate.

### Scanning electron microscopy of *C. albicans* biofilms.

In order to observe the effect of 9029936 on biofilm formation, *C. albicans* SC5314 cells at a concentration of 1 × 10^6^ cells/ml in RPMI 1640 were used to seed in the presence or absence of 9029936 at 5 µM in a 24-well plate containing a round glass slide at the bottom of each well. Plates were incubated at 37°C for 24 h, washed, and fixed as previously described (50). Briefly, biofilms were fixed with a solution of glutaraldehyde (2.5% [wt/vol])–0.1 M sodium cacodylate buffer at pH 7.4) for 2 h at 37°C. Biofilms were then treated with osmium tetraoxide (1% [wt/vol])–0.1 M sodium cacodylate buffer at pH 7.4 for 2 h at room temperature. Samples were then rinsed and soaked in a step gradient of ethanol (30%, 50%, 70%, 90%, and 100%) for 10 min per gradient. Samples were dried overnight and coated with a 60:40 gold-palladium alloy combination and imaged using a scanning electron microscope.

### Cytotoxicity assays.

We used a cell-based assay as an alternative to animal testing to determine the initial toxicities of the selected compounds (51, 52). Briefly, human hepatocellular carcinoma (HepG2) cells (American Type Culture Collection [ATCC] HB-8065) were maintained in minimum essential medium (Gibco-Life Technologies, Inc., Grand Island, NY, USA) supplemented with 10% fetal bovine serum, 1 mM sodium pyruvate (Gibco), 1× minimum essential medium amino acid solution (Sigma), 100 IU/ml penicillin, and 100 mg/ml streptomycin (Cellgro). The monolayers of cells were detached using 1 × trypsin-EDTA (Gibco) and centrifuged at 500 × *g* for 7 min at 4°C. The cell count was adjusted to 5 × 10^5^ cells/ml in supplemented minimum essential medium (twice), and 100 μl of the cell suspension was added to each well of white-bottom, 96-well microtiter plates containing 100 μl of serial 2-fold dilutions of the small-molecule compounds (160 to 1.56 μM) or the DMSO control. The plates were incubated for 24 h at 37°C, and the number of viable cells was determined in the CellTiter-Glo luminescent cell viability assay (Promega, Madison, WI). From these data, the CC_50_ values, defined as the concentration of each compound leading to 50% inhibition of cell viability, were calculated by using Prism (GraphPad Software, Inc.).

### Determination of the *in vivo* activities of the leading compounds in murine models of hematogenously disseminated candidiasis and oral candidiasis.

All animal experiments were performed following NIH guidelines and in accordance with institutional regulations (IACUC) in an AAALAC-certified facility at UTSA. Animals were allowed a 1-week acclimatization period before experiments were started. Mice were randomly distributed in different cages and assigned to the different treatment arms, and persons monitoring the animals were not blinded as to the identity of different groups. As per IACUC guidelines for power analyses, we used data from similar past experiments to estimate the (lower) number of animals to be used in experiments required for significance.

To prepare the initial infecting inocula, cultures of strain SC5314 were grown overnight in YPD broth at 25°C. Cells were harvested by centrifugation and washed three times with sterile saline. Cells were counted using a hemocytometer, and appropriate dilutions of the cells were made in sterile saline for injection. Confirmation of the number and viability of cells present in the infecting inocula was performed via plate counts.

For the hematogenously disseminated candidiasis model, a final volume of 200 µl containing 3.5 × 10^5^ cells of *C. albicans* strain SC5314 was injected via the lateral tail vein into 6- to 8-week-old female BALB/c mice. Groups of mice (*n* = 8) were treated intraperitoneally with 0.5 ml of an 0.8-mg/ml solution (corresponding to approximately 20 mg/kg of body weight) of either compound 9029936 or 7977044 diluted in 4% DMSO (prepared in saline for injection) starting 2 days prior to infection, with treatment continuing once a day until 7 days postinfection, at which time the treatment was stopped. A control group was on the same schedule but received vehicle-only injections. To determine the survival curves, days on which the mice died were recorded; moribund mice were humanely euthanized and recorded as dying the following day. Survival data and differences between groups (treated versus untreated) were analyzed using the Kaplan-Meier and log rank tests. Analyses were performed using Prism (GraphPad Software, Inc.).

We also performed a series of time-scheduled sacrifices for determination of fungal organ burdens. Briefly, one group of mice (*n* = 16) was treated with compound 9029936 as described above, whereas another group (untreated control; *n* = 16) was injected with vehicle only. All animals were infected intravenously with 2.5 × 10^5^ *C. albicans* SC5314 cells, and four animals from each group were then sacrificed at 1, 2, 3, and 6 days postinfection. For both treated and control (untreated) animals, fungal loads were determined by removing and homogenizing one kidney, the brain, and the spleen from each mouse at the time of sacrifice and plating dilutions onto Sabouraud dextrose agar (Becton, Dickinson, Franklin Lakes, NJ) plates containing ampicillin. After 24 h of incubation, colonies were counted to determine the CFU per gram of tissue. For analyses of fungal organ burdens, CFU data were converted into logarithmic values, and the nonparametric Mann-Whitney test was used to determine statistical significance between treated and untreated groups.

For histological analyses, kidneys were removed at the time of death or sacrifice, fixed in 10% buffered formalin, embedded in paraffin, sectioned, and stained with Grocott-Gomori methenamine-silver (GMS) prior to microscopic evaluation for determination of fungal morphology within infected tissues.

The *in vivo* efficacy of the 9029936 compound was also determined using the murine model of oropharyngeal candidiasis, mostly as previously described by the Filler group (40, 41), with slight modifications. Briefly, groups of 6- to 8-week-old female BALB/c mice were immunosuppressed by subcutaneous injection with 225 mg/kg of cortisone acetate (Sigma-Aldrich) on day 1 preinfection and days 1 and 3 postinfection. For infection, each mouse was anesthetized by an intramuscular injection with 50 μl of 2 mg/ml chlorpromazine chloride and inoculated sublingually for 75 min with a swab saturated with 1 × 10^6^ *C. albicans* yeast cells (from cultures grown overnight in YPD broth at 25°C) per ml of saline. For treatment, a 0.8-mg/ml solution of the test compound was prepared as described above in saline for injection. One group of mice (*n* = 5) received vehicle only and served as the untreated control, and a second group of mice (*n* = 5) received daily intraperitoneal injections of compound 9029936 in 0.5 ml of saline (for a dose equivalent to 20 mg/kg). Treatments started 2 days prior to infection. Macroscopic evaluation of the infection was used to generate a clinical score from 0 to 4 on the basis of the extent and severity of the tongue lesions (53), with 0 denoting a healthy tongue surface and 4 the most severe stage (white patches covering close to 100% of the surface). Scoring data were compared using the nonparametric Mann-Whitney test (one-tailed, based on the prediction that treatment would have beneficial effects, and assuming equal variances). After 3 days of infection, mice were sacrificed and their tongues were excised, embedded in paraffin, sectioned, and stained with GMS for visualization of fungal elements.
