# Supplementary figures and images for: Development of Anti-Virulence Approaches for Candidiasis via a Novel Series of Small-Molecule Inhibitors of Candida albicans Filamentation
Source: mBio. 2017 Dec 5;8(6):e01991-17. doi: 10.1128/mBio.01991-17 (PMC5717394; doi:10.1128/mBio.01991-17)

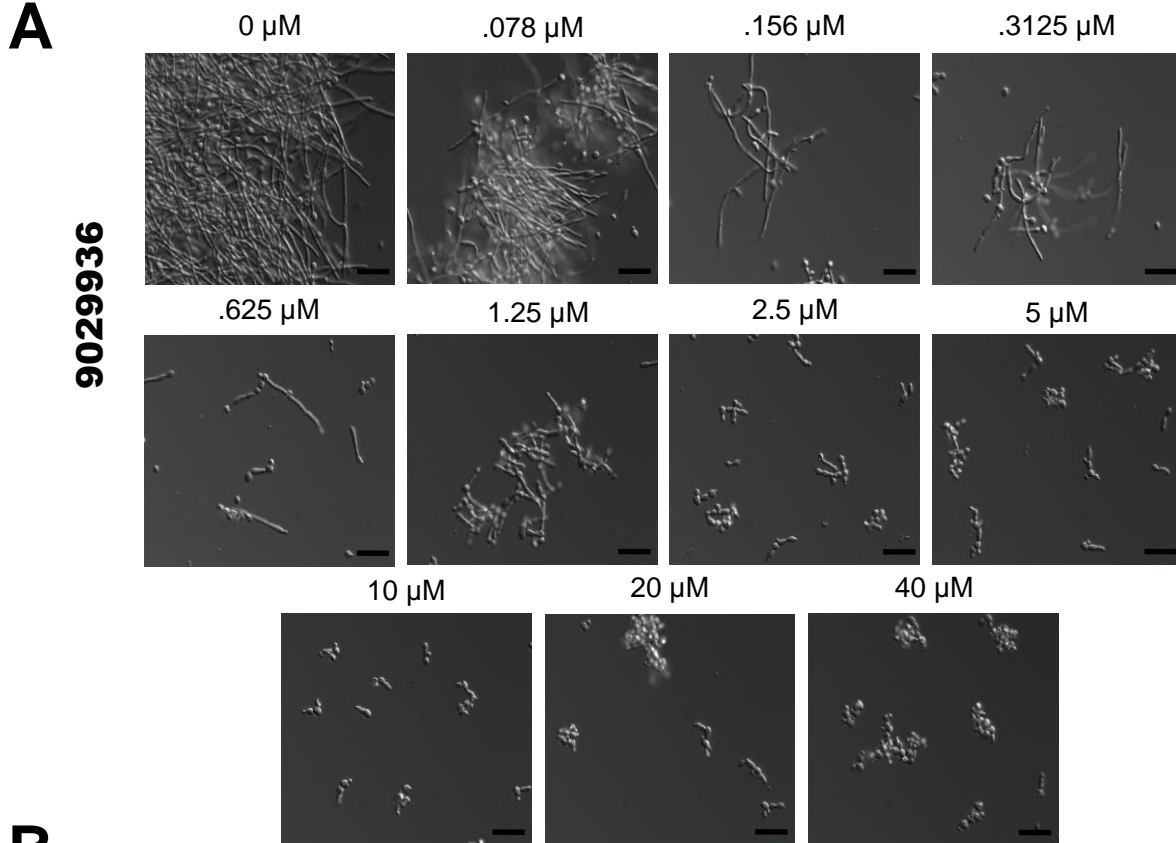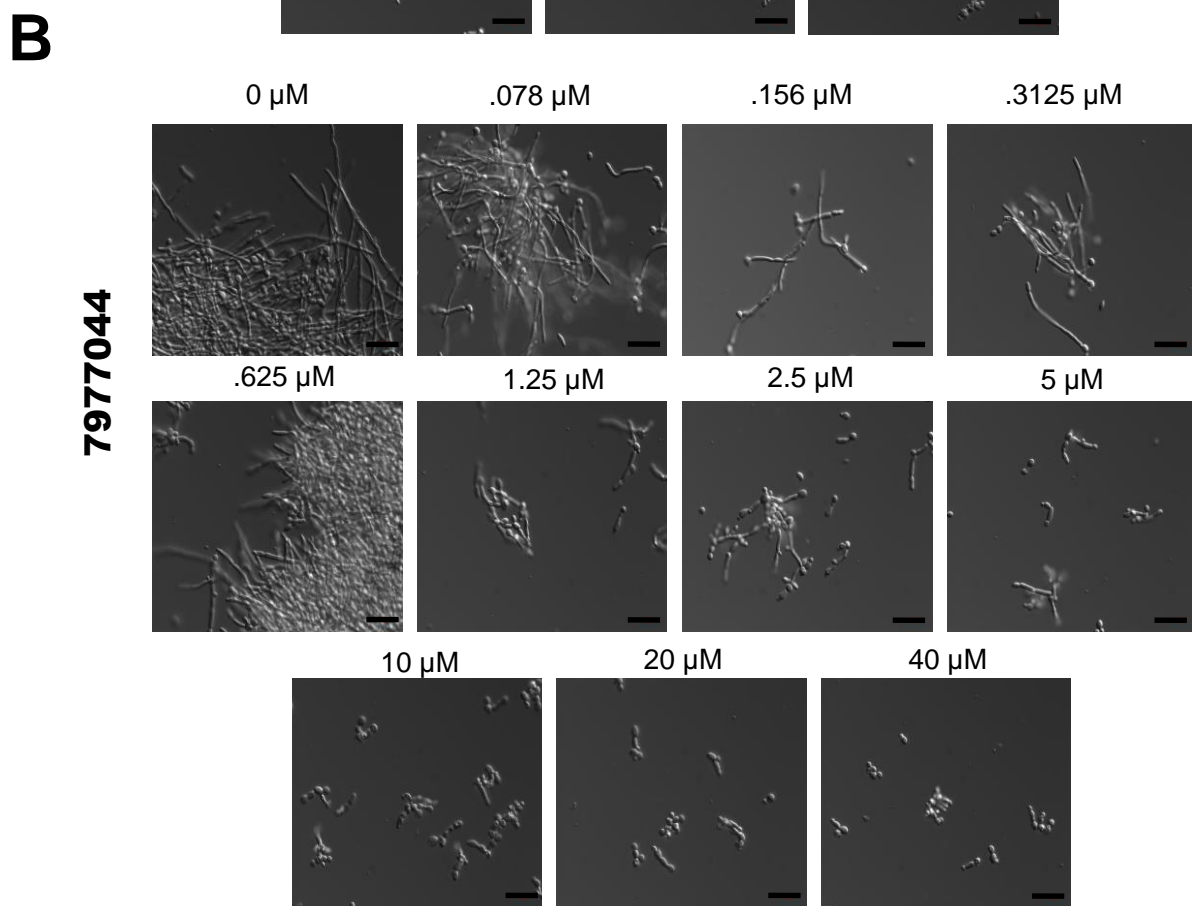

Supplement: FIG S1 [file mbo006173629sf1.pdf]

9029936

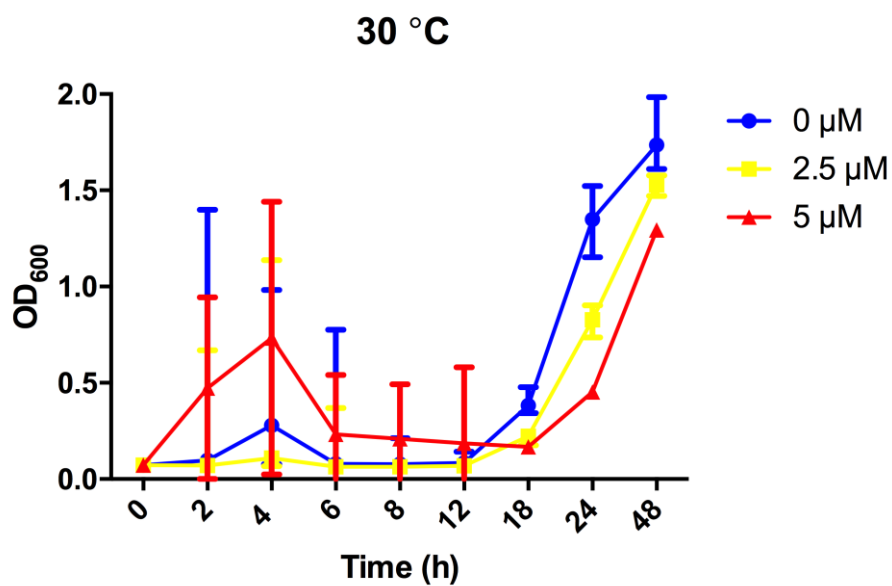

7977044

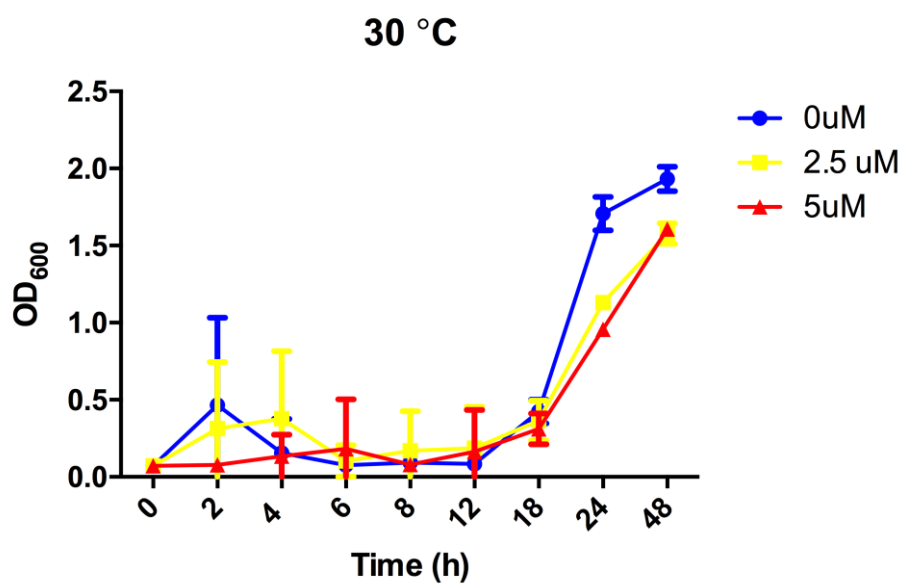

Supplement: FIG S2 [file mbo006173629sf2.pdf]

Untreated

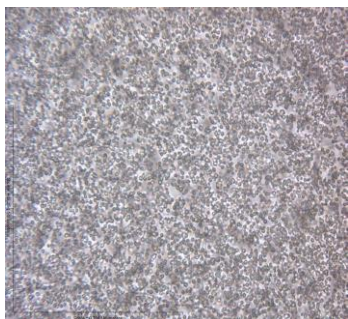

25  $\mu$ M DTPA

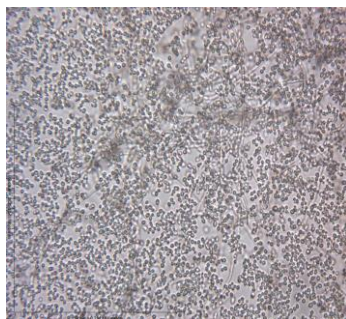

50  $\mu$ M DTPA

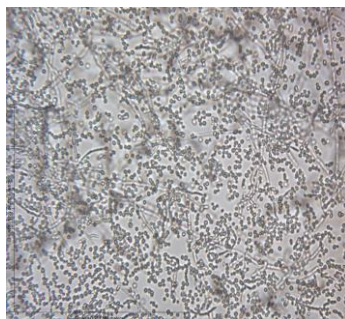

100  $\mu$ M DTPA

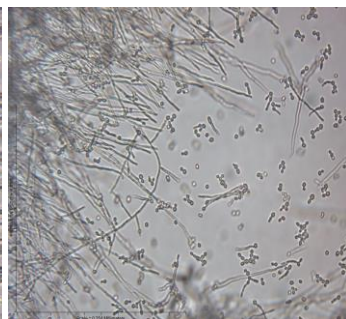

100  $\mu$ M DTPA +  
40  $\mu$ M 9029936

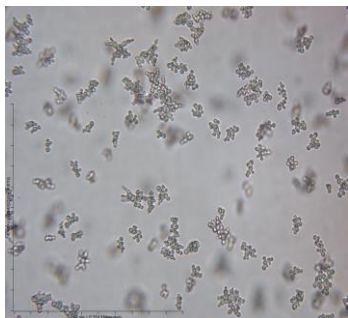

100  $\mu$ M DTPA +  
20  $\mu$ M 9029936

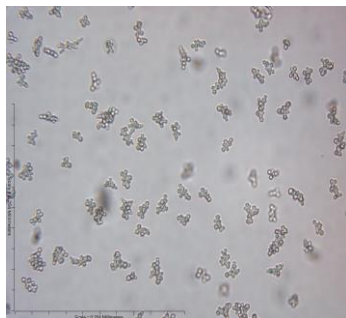

100  $\mu$ M DTPA +  
10  $\mu$ M 9029936

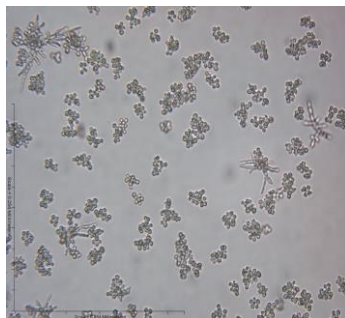

100  $\mu$ M DTPA +  
5  $\mu$ M 9029936

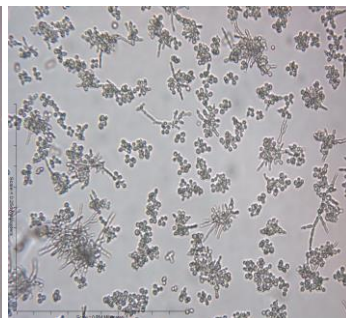

Supplement: FIG S3 [file mbo006173629sf3.pdf]

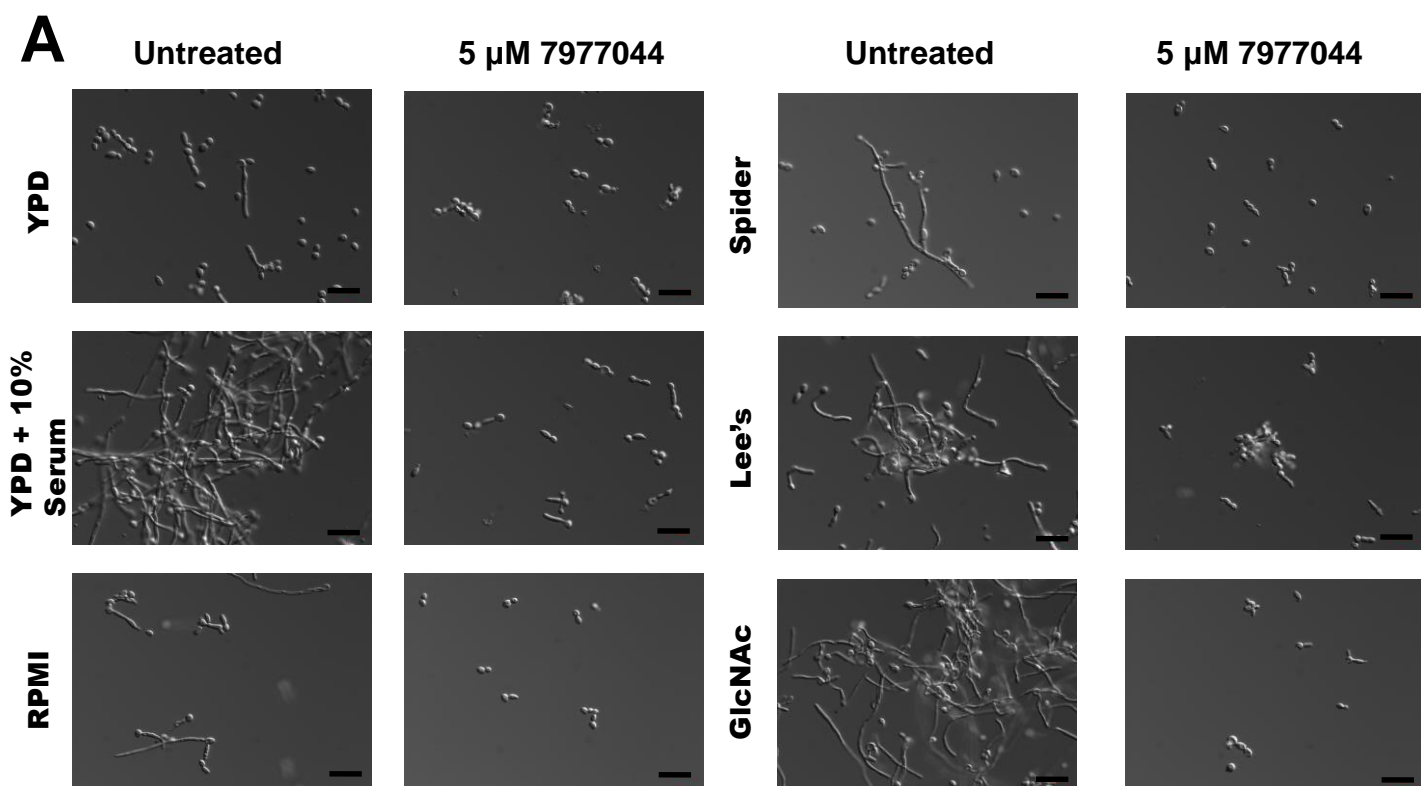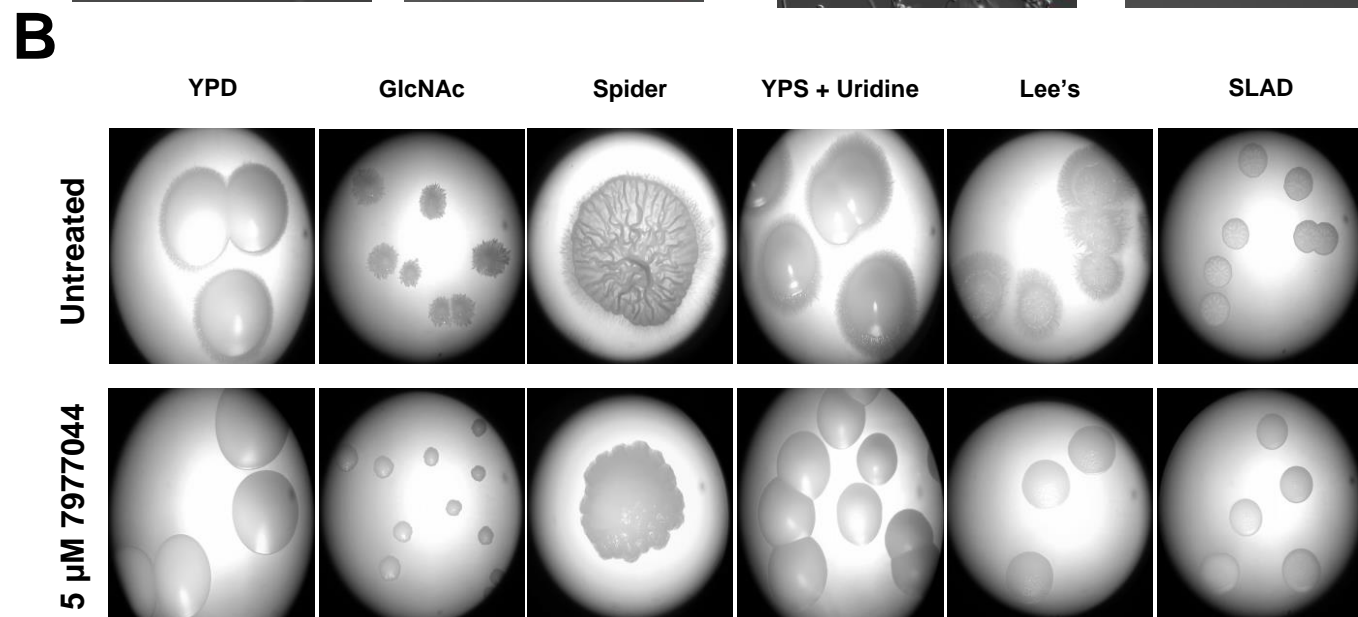

Supplement: FIG S4 [file mbo006173629sf4.pdf]

## A Dose Response for Biofilm Inhibition

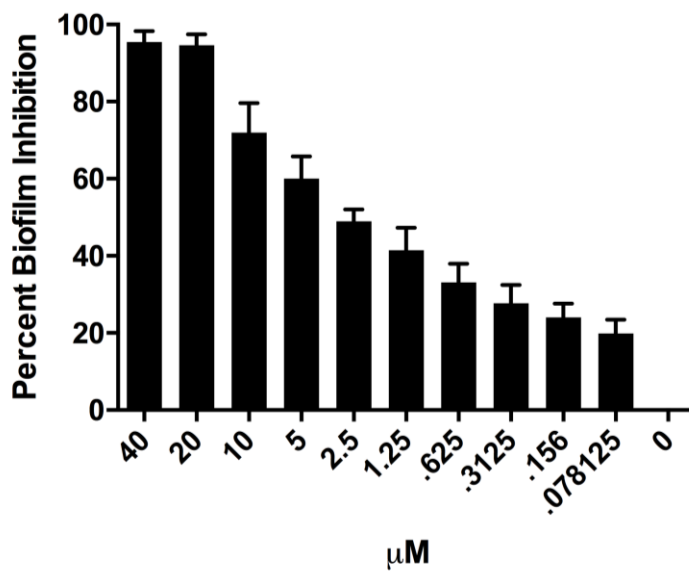

## Biofilm Kinetics

B

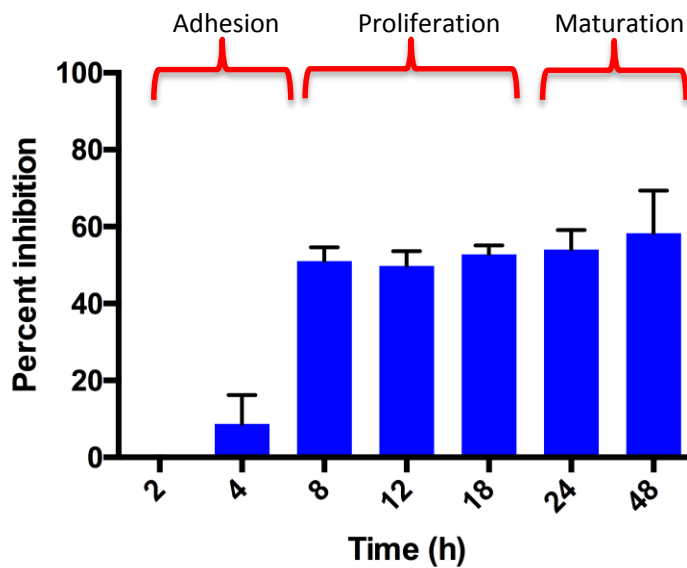

Supplement: FIG S5 [file mbo006173629sf5.pdf]

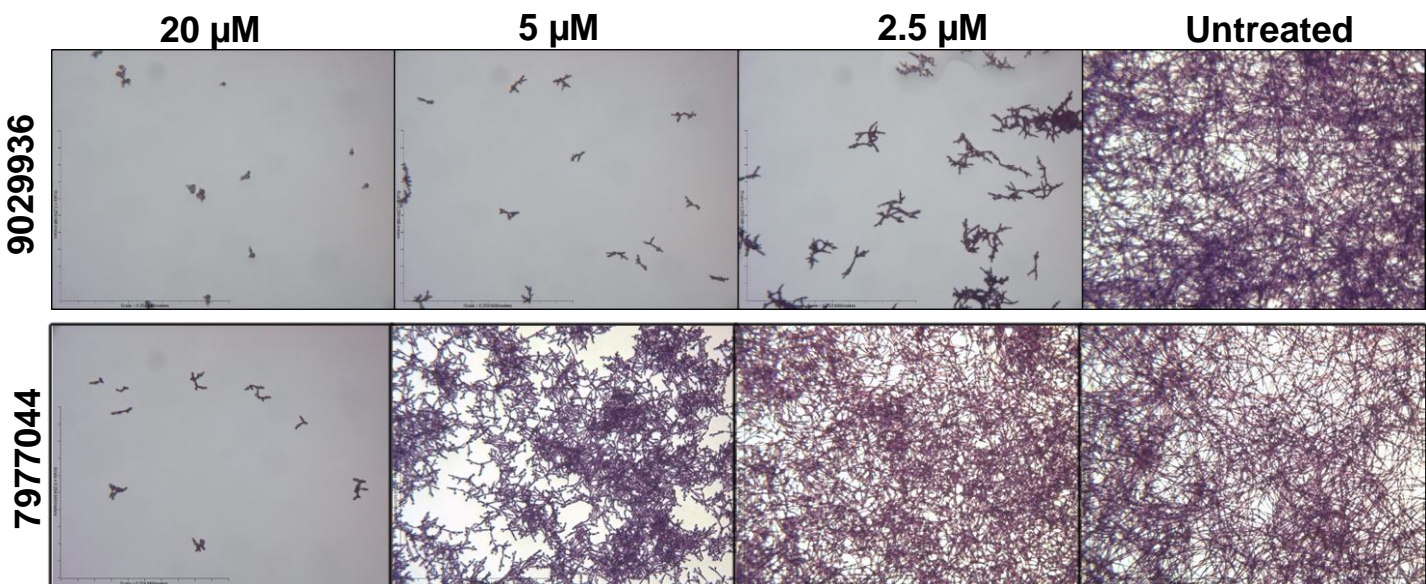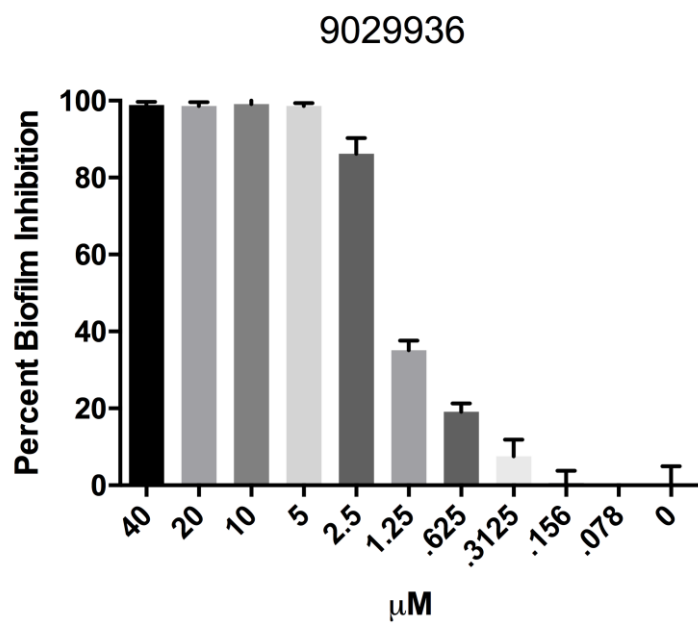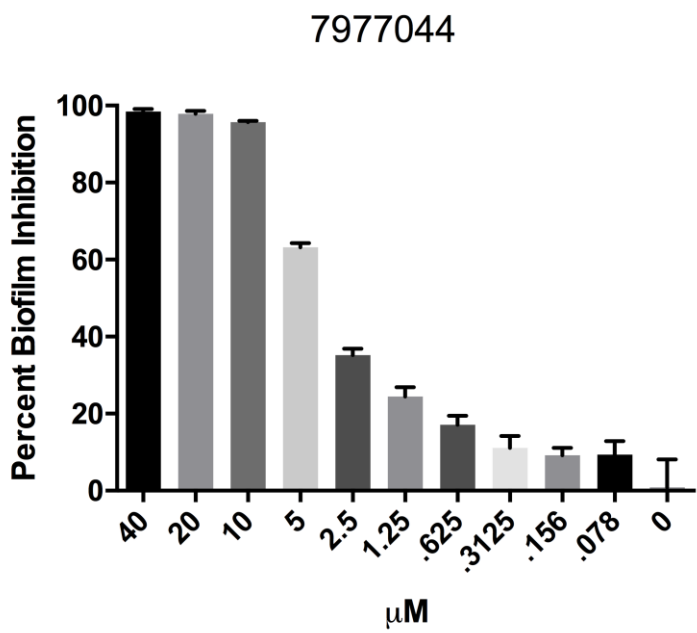

Supplement: FIG S6 [file mbo006173629sf6.pdf]

**Pre-formed biofilms**

**9029936**

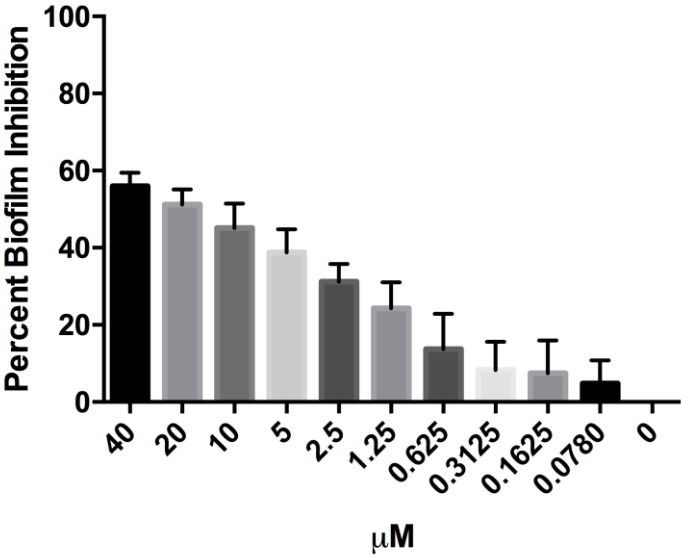

**7977044**

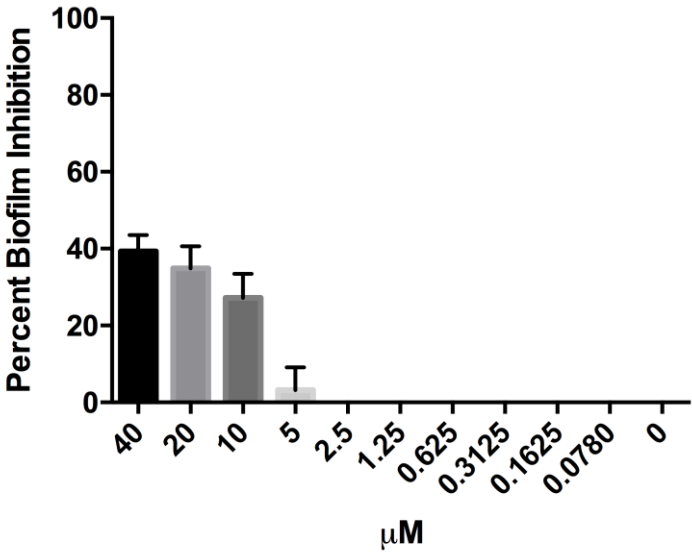

Supplement: FIG S7 [file mbo006173629sf7.pdf]

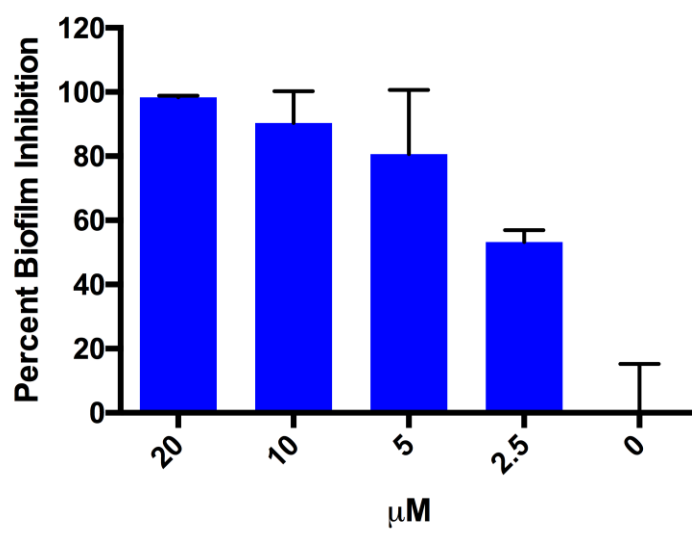

Supplement: FIG S8 [file mbo006173629sf8.pdf]

A

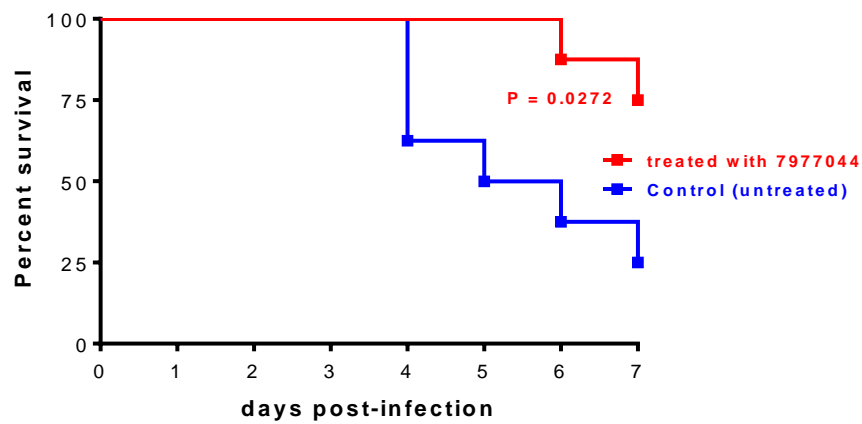

B

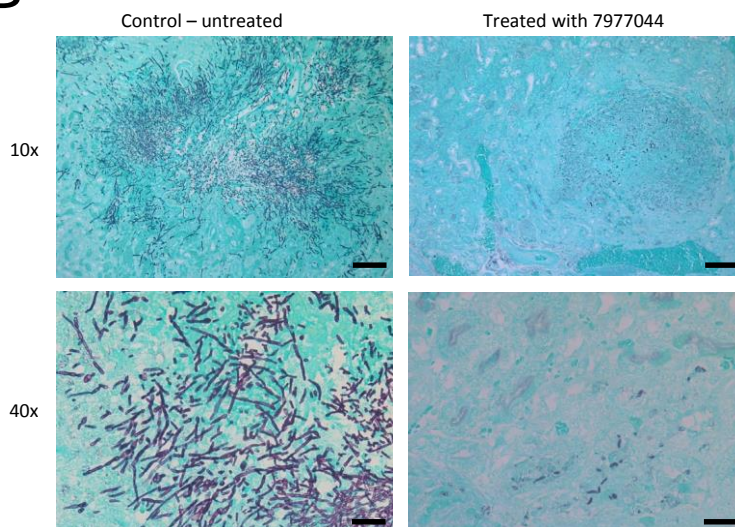

Supplement: FIG S9 [file mbo006173629sf9.pdf]

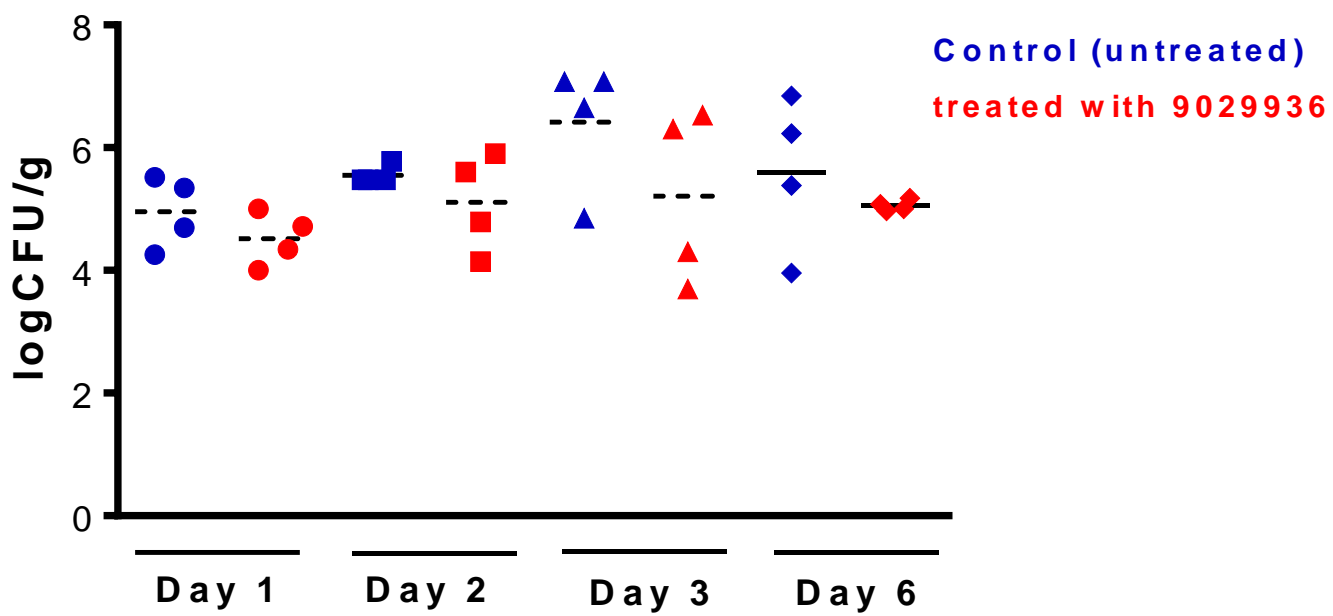

Supplement: FIG S10 [file mbo006173629sf10.pdf]
